# Ephemeral Diabetes After COVID-19 Vaccination

**DOI:** 10.1210/jcemcr/luae228

**Published:** 2024-12-13

**Authors:** Sarah-Ålivia Mänd, Åke Sjöholm

**Affiliations:** Department of Internal Medicine, Division of Endocrinology and Diabetology, Gävle Hospital, Gävle SE-80324, Sweden; Department of Internal Medicine, Division of Endocrinology and Diabetology, Gävle Hospital, University of Gävle, Gävle SE-80324, Sweden

**Keywords:** islet, COVID-19, vaccination, diabetes

## Abstract

We report a case of new-onset, nonautoimmune, nonketotic, and noninsulinopenic type 2-like diabetes in a previously normoglycemic middle-aged man debuting after vaccination against COVID-19. This was not a mild or short-lived glucose intolerance, but severe and long-standing hyperglycemia with a high glycated hemoglobin level. However, the course of the diabetes was highly atypical and surprising in that it spontaneously disappeared after a few months and did not recur despite the patient being off all antidiabetic drugs for several months and without any changes in body weight or lifestyle. The mechanisms by which severe diabetes unfolded and later remitted in this patient remain elusive. Nonetheless, and notwithstanding whether or not there was a cause-and-effect relation between the vaccinations and his diabetes, the highly atypical course of spontaneously remitting nonautoimmune diabetes lends itself to mechanistic efforts aimed at understanding the biology and pathophysiology of insulin-producing β cells in health and disease. This case report should not be construed as vaccine skepticism or deter anyone, especially with diabetes/obesity, from vaccination against COVID-19. However, it calls for increased vigilance among health care providers for unusual and unexpected metabolic effects of COVID-19 and its vaccines.

## Introduction

After the onset of the COVID-19 pandemic, several case reports appeared describing new-onset diabetes, both type 1- and type 2-like, in close connection with the SARS-CoV-2 infection. It is, however, virtually impossible to rule out that these persons would have acquired their diabetes anyway and that it just coincided with the SARS-CoV-2 infection (ie, the virus would not be a causative agent but rather an innocent bystander).

Systemic reviews have shown conflicting results as to whether there is any increased risk of new-onset diabetes after SARS-CoV-2 infection [1]. In many cases, there is a brief transient hyperglycemia at diabetic or prediabetic levels, probably caused by the cytokine storm caused by SARS-CoV-2 infection, which soon resolves. The interpretation of any relation between SARS-CoV-2 infection and new-onset diabetes is further confounded by the abundant use of potent glucocorticoids, known diabetogenic hormones, in patients with severe COVID-19.

Conversely, patients with known diabetes or obesity, or with hyperglycemia at admission, were found to be at increased risk not only to be infected by SARS-CoV-2, but also showing more complications and increased mortality compared to nonobese, nondiabetic individuals [1]. Thus, patients with diabetes—especially with obesity—were soon recognized as a high-risk group for COVID-19 and were strongly advised to receive early vaccination.

There are also anecdotal reports in the literature of cases in which diabetes developed after vaccination against COVID-19. However, systemic reviews have failed to find such an association in large populations [2, 3]. Nonetheless, these epidemiological findings at group level do not exclude the possibility that such a causal relationship may occur in individual patients.

## Case Presentation

A 58-year-old male patient, who in 2018 had suffered a cerebellar bleed, leaving him severely disabled, and with obesity, dyslipidemia, and hypertension, underwent a vaccination program against COVID-19.

In May 2021, the patient received his first injection of COVID-19 vaccine (Pfizer, lot Ex6537), followed by a second injection (Pfizer, lot FD6840) in June 2021, and a third injection (Moderna, lot 3006273) in January 2022.

In February 2022, approximately 1 week after the third and final COVID-19 vaccination, the patient developed classic symptoms of diabetes with polydipsia and polyuria.

The patient had been on the same oral medications for several years, with no change to date. They consist of: metoprolol 100 mg every day, felodipin 5 mg every day, enalapril 20 mg every day, doxazosin 2 mg twice daily, spironolactone 25 mg every day, and atorvastatin 10 mg every day. No other vaccines had been given and there was no use at all of steroids or other diabetogenic agents.

## Diagnostic Assessment

At a check-up for these symptoms, the patient had a fasting plasma glucose of 27.5 mmol/L (reference 4-6 mmol/L) (495 mg/dL [reference 72-108 mg/dL]) and a level of glycated hemoglobin (HbA_1c_) of 136 mmol/mol (reference 31-46 mmol/mol) (14.6% [reference 5.0-6.4%]). C-peptide was 1.5 nmol/L (reference 0.37-1.5 nmol/L) (4.5 ng/mL [ref. 1.1-4.5 ng/mL]). Prior to the vaccinations, the patient did not have any dysglycemia with a fasting plasma glucose of 5.6 mmol/L (101 mg/dL) in February 2021. In March 2022, the patient's HbA_1c_ level was 126 mmol/mol (13.7%). Although we have not yet performed an oral glucose tolerance test, we did not find any causes of inaccurate HbA_1c_ (eg, anemia), and professional glucose sensors were used throughout.

## Treatment

After being diagnosed with type 2 diabetes in February 2022, the patient was started on metformin and later also empagliflozin with good effect on glycemia. The patient decided, however, for unknown reasons, to discontinue his medications after 5 months. Surprisingly, he found himself normoglycemic despite being off the drugs for 5 months. He also did not change his lifestyle in any way (eg, diet, exercise, body weight).

## Outcome and Follow-up

The patient came for a scheduled visit to the diabetes outpatient clinic in December 2022. Surprisingly, however, his diabetes had disappeared spontaneously despite being off all treatment for 5 months.

At this time, the patient had a fasting plasma glucose of 5.6 mmol/L (101 mg/dL) and an HbA_1c_ of 39 mmol/mol (5.7%). C-peptide was 2.9 nmol/L (8.76 ng/mL). Analyses of antibodies against known β-cell autoantigens (glutamic acid decarboxylase, insulinoma antigen 2, zinc transporter 8) were all negative. Thus, he had a completely normal glucose regulation, not even close to prediabetes.

Since July 2022, the patient is off all antidiabetic drugs and remains normoglycemic to this day. Despite sometimes consuming large meals with rapid carbohydrates (eg, pasta), his postprandial plasma glucose levels rarely exceed 6 mmol/L (108 mg/dL). The patient's body weight (105 kg [231 lb]) did not change materially over the entire time course (diagnosis, remission, currently).

The case was reported to the Swedish Medical Products Agency as a possible side effect of the vaccinations.

## Discussion

We share a case of new-onset, nonautoimmune, nonketotic, and noninsulinopenic type 2-like diabetes debuting after vaccination against COVID-19.

This was not a mild or short-lived glucose intolerance, but severe and long-standing hyperglycemia as reflected by the high HbA_1c_ level. However, the course of the diabetes was highly atypical and surprising in that it spontaneously disappeared after a few months and did not recur despite the patient being off all antidiabetic drugs for several months and without any changes in body weight or lifestyle.

This is very much different from the natural course of conventional type 2 diabetes, which is a progressive disease characterized by slow but relentless loss of insulin-producing β-cell functional mass over time [4]. Long-term remission, at least temporarily, of type 2 diabetes may occur in individuals who change their lifestyle dramatically, usually related to massive weight loss that is also seen after bariatric/metabolic surgery or after long-term treatment with glucagon-like peptide 1 receptor agonists [5]. This patient had none of this.

The mechanisms by which severe diabetes unfolded and later remitted in this patient remain elusive. Nonetheless, and notwithstanding whether or not there was a cause-and-effect relation between the vaccinations and his diabetes, the highly atypical course of spontaneously remitting nonautoimmune diabetes lends itself to mechanistic efforts aimed at understanding the biology and pathophysiology of insulin-producing β cells in health and disease.

This case report should not be construed as vaccine skepticism or deter anyone, especially with diabetes/obesity, from vaccination against COVID-19. However, it calls for increased vigilance among health care providers for unusual and unexpected metabolic effects of COVID-19 vaccines.

## Learning Points

- We describe a case of diabetes debuting after vaccination against COVID-19.
- This was a severe and long-standing diabetes as reflected by the high glycated hemoglobin level.
- The course of the diabetes was, however, highly atypical in that it had disappeared after a few months and did not recur.
- The patient remains to this day, November 2024, completely glucose tolerant without any antidiabetic medications for 2 years and 4 months or lifestyle modifications. This case report should not be construed as vaccine skepticism or deter anyone, especially with diabetes/obesity, from vaccination against COVID-19.
- The case calls, however, for increased vigilance among health care providers for unusual and unexpected metabolic effects of Covid-19 and its vaccines.
- The duration of disease remission in this patient will be monitored by regular check-ups of his glucose tolerance.

## Contributors

Å.S. cared for the patient and wrote and edited the manuscript. S-Å.M. wrote and edited the manuscript. All authors approved the final version of the manuscript. Å.S. is the guarantor of this work and, as such, had full access to all the data in the study and takes responsibility for the integrity of the data and the accuracy of the data analysis.

## Data Availability

Restrictions apply to the availability of some or all data generated or analyzed during this study to preserve patient confidentiality or because they were used under license. The corresponding author will on request detail the restrictions and any conditions under which access to some data may be provided.

## Abbreviation

HbA_1c_: glycated hemoglobin
